# Blood Outgrowth and Proliferation of Endothelial Colony Forming Cells are Related to Markers of Disease Severity in Patients with Pulmonary Arterial Hypertension

**DOI:** 10.3390/ijms19123763

**Published:** 2018-11-27

**Authors:** Josien Smits, Dimitar Tasev, Stine Andersen, Robert Szulcek, Liza Botros, Steffen Ringgaard, Asger Andersen, Anton Vonk-Noordegraaf, Pieter Koolwijk, Harm Jan Bogaard

**Affiliations:** 1 Amsterdam UMC, VU University Medical Center, Department of Pulmonary Diseases, Amsterdam Cardiovascular Sciences (ACS), De Boelelaan 1118, 1081 HV Amsterdam, The Netherlands; aj.smits1@vumc.nl (J.S.); r.szulcek@vumc.nl (R.S.); l.botros@vumc.nl (L.B); a.vonk@vumc.nl (A.V.-N.); 2 Amsterdam UMC, VU University Medical Center, Department of Physiology, Amsterdam Cardiovascular Sciences (ACS), De Boelelaan 1108, 1081 HV Amsterdam, The Netherlands; dimitasev@gmail.com (D.T.); p.koolwijk@vumc.nl (P.K.); 3 Aarhus University Hospital, Department of Cardiology, Palle Juul-Jensens Boulevaard 99, 8200 Aarhus N, Denmark; stineandersen@clin.au.dk (S.A.); asger.andersen@clin.au.dk (A.A.); 4 Aarhus University Hospital, MR Centre, Palle Juul-Jensens Boulevaard 99, 8200 Aarhus N, Denmark; steffen@clin.au.dk

**Keywords:** pulmonary hypertension, pulmonary vascular disease, endothelial colony forming cell, endothelial progenitor cell

## Abstract

In pulmonary arterial hypertension (PAH), lung-angioproliferation leads to increased pulmonary vascular resistance, while simultaneous myocardial microvessel loss contributes to right ventricular (RV) failure. Endothelial colony forming cells (ECFC) are highly proliferative, angiogenic cells that may contribute to either pulmonary vascular obstruction or to RV microvascular adaptation. We hypothesize ECFC phenotypes (outgrowth, proliferation, tube formation) are related to markers of disease severity in a prospective cohort-study of 33 PAH and 30 healthy subjects. ECFC were transplanted in pulmonary trunk banded rats with RV failure. The presence of ECFC outgrowth in PAH patients was associated with low RV ejection fraction, low central venous saturation and a shorter time to clinical worsening (5.4 months (0.6–29.2) vs. 36.5 months (7.4–63.4), *p* = 0.032). Functionally, PAH ECFC had higher proliferative rates compared to control in vitro, although inter-patient variability was high. ECFC proliferation was inversely related to RV end diastolic volume (*R*^2^ = 0.39, *p* = 0.018), but not pulmonary vascular resistance. Tube formation-ability was similar among donors. Normal and highly proliferative PAH ECFC were transplanted in pulmonary trunk banded rats. While no effect on hemodynamic measurements was observed, RV vascular density was restored. In conclusion, we found that ECFC outgrowth associates with high clinical severity in PAH, suggesting recruitment. Transplantation of highly proliferative ECFC restored myocardial vascular density in pulmonary trunk banded rats, while RV functional improvements were not observed.

## 1. Introduction

In pulmonary arterial hypertension (PAH) angio-proliferation, inflammation and vasoconstriction of the small arteries of the lungs progressively increase pulmonary vascular resistance (PVR) [1,2]. The right ventricle (RV) responds to an augmented afterload with proportional hypertrophy and microvascular adaptation in an attempt to preserve RV ejection fraction (RVEF) [3]. Maintenance of RV function is highly critical for the survival of PAH patients. When despite treatment terminal right heart failure develops, lung-transplantation remains the only therapeutic option [4].

In response to tissue damage, inflammation or hypoxia, endothelial progenitor cells (EPC) are recruited to the peri-vasculature following a gradient of cytokines and chemokines. EPC arise from the mononuclear cell fraction (MNC) of blood, and as a class EPC contain several cell populations, including late outgrowth endothelial colony forming cells (ECFC) [5]. As opposed to early outgrowth cells, ECFC appear late in culture (7–28 days) in the form of (multiple) expansive monoclonal colonies [6]. ECFC do not express immune cell markers (CD14, CD45) as opposed to early outgrowth EPC [7]. On the contrary, they express markers indicating a myeloid stem cell lineage (CD34, c-kit) and resemble endothelial cells in morphology and EC marker expression (i.e., CD144, CD31, VEGFR-2, VWF) [5,8]. It is generally believed that under physiological conditions, ECFC maintain vascular hemostasis by replacement of damaged endothelium and vascularization of ischemic tissue [9,10,11].

Previous studies have shown altered numbers of circulating EPC in PAH and have also suggested abnormal ECFC behavior [12,13,14,15]. The different studies have provided contradictory results, however, and the exact role of ECFC in PAH pathobiology remains debated. PAH is a vascular disease characterized by an imbalance in vascular damage and repair responses in both heart and lungs, and ECFC may exert opposite effects in these organs. In the stressed heart, ECFC may be capable of inducing neovascularization and thereby facilitate adaptive hypertrophic cardiac remodeling. In the lungs, ECFC could maintain integrity of damaged endothelium, preventing vascular rarefaction, but at the same time, abnormal ECFC behavior may also be a key component of occlusive angio-proliferative vascular remodeling.

Previous research has suggested that transplantation of autologous EPC has a beneficial effect on hemodynamic and functional parameters of PAH patients [16,17], and in models of experimental pulmonary hypertension [18,19,20,21]. On the other hand, in vitro studies have shown signs of dysfunction in ECFC of PAH patients [12,13,14,15] suggesting these cells may mediate disease progression [22]. Until now, ECFC have not been studied in the context of lung or cardiac vascular remodeling. Understanding the role of ECFC in PAH may ultimately result in new biomarkers to monitor PAH, in new therapeutic interventions aimed at changing the number of ECFC, or in the use of ECFC as a vector for targeted therapy [23,24].

In this prospective cohort study, we aimed to investigate the clinical significance of ECFC (dys-) function in PAH. ECFC were isolated from 33 patients with PAH and from 30 healthy control subjects. Blood outgrowth, proliferation and tube formation were quantified. Our hypothesis was that because ECFC participate in pulmonary vascular and/or right heart remodeling, ECFC functional read-outs will correlate with clinical characteristics and markers of disease severity. PAH ECFC functional read-outs were compared to clinical data, including right heart catheterization, cardiac magnetic resonance (CMR) imaging and time to clinical worsening (TTCW) over a period of 3 years. Because ECFC proliferative capacity correlated inversely with RV function but not PVR, we subsequently transplanted PAH ECFC in rats with chronic RV failure induced by pulmonary trunk banding (PTB) to determine effects on RV function and vascular density.

## 2. Results

### 2.1. PAH Patient Characteristics and ECFC Outgrowth

**Figure 1:**
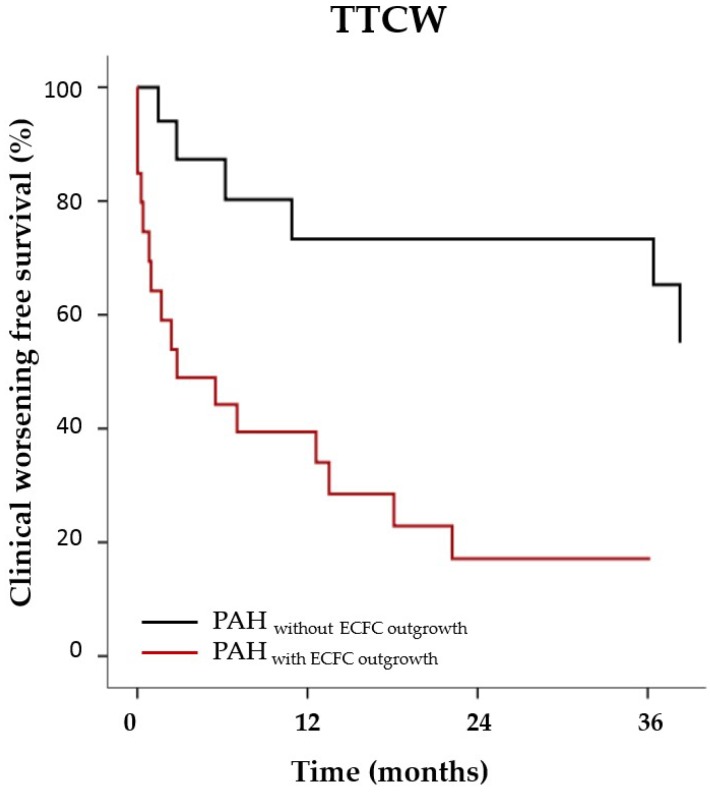
PAH patients with outgrowth of ECFC are more likely to experience early clinical worsening. Kaplan Meijer curves of PAH patients with outgrowth of ECFC (red, *n* = 21) without outgrowth of ECFC (grey, *n* = 12) over a period of 36 months (*x*-axis). Cox-regression corrected for age; *p* = 0.032.

ECFC were isolated from 33 patients with PAH and 30 healthy control subjects. Out of 33 PAH patients, 21 had outgrowth of at least one ECFC colony (64%). Likewise, 20 control subjects gave outgrowth to ECFC (67%). All ECFC-lineages expressed mature endothelial cell markers (Figure S1). The quantity of single outgrown colonies was neither related to peripheral oxygen concentration nor to the presence of PAH disease (Figure S2). Characteristics of PAH patients with and without outgrowth of ECFC colonies are presented in Table 1. PAH patients with ECFC outgrowth were younger and had significantly lower RVEF compared to patients without ECFC outgrowth. Patients with outgrowth of ECFC experienced significantly earlier clinical worsening defined by hospital admittance, a decrease in exercise capacity, and the start of invasive treatment with prostaglandin or death; (cox regression of TTCW corrected for age, for PAH with outgrowth for PAH without outgrowth, *p* = 0.032, see Figure 1). Mean age of control ECFC subjects was 29 ± 7 years. There was no age difference between healthy control subjects with and without outgrowth of ECFC colonies (Table S1).

### 2.2. Quantification of PAH ECFC Proliferation and Tube Formation

#### 2.2.1. Proliferation

**Figure 2:**
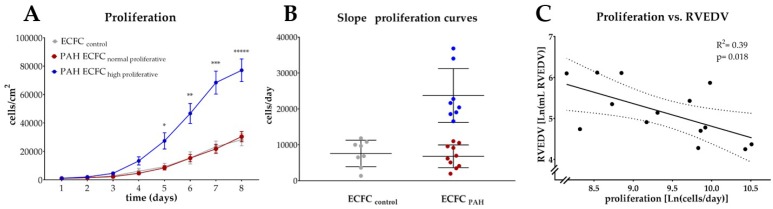
PAH ECFC have higher proliferative rates compared to ECFC from healthy control subjects, while ECFC proliferation is related to RVEDV. (**A**) Quantification of cell density (*y*-axis, cells/cm^2^) of ECFC of healthy subjects (*n* = 8, grey) and PAH donors (*n* = 18, red and blue) over time (*x*-axis, days) indicates PAH ECFC proliferation is significantly higher compared to ECFC from healthy subjects, due to a subset of highly proliferative of PAH ECFC (*n* = 8, blue). Two way repeated measurements ANOVA, *p* < 0.0001. Mean with standard error of mean (SEM) is shown. (**B**) Quantification of the steepness of individual growth curves (*y*-axis, cells/day) indicates high proliferative PAH ECFC exceed the mean steepness of the growth curve of control ECFC by >2SD, where mean with SD is shown. (**C**) Individual steepness of the growth curve of PAH ECFC is correlated to RVEDV (*y*-axis, Ln (RVEDV, mL), linear regression, *R^2^* = 0.39, *p* = 0.018, *n* = 14. 95% confidence interval (CI) is shown of Ln transformed data.

ECFC cultures for further experimentation were available from 18 patients. PAH ECFC had a higher proliferative rate, and reached confluence sooner than ECFC from normal subjects (two way repeated measurements ANOVA, *p*-interaction = 0.018, *t* = 8 days (Figure S3). The overall higher proliferative rate could be attributed to a subset of PAH patients exhibiting a significantly increased proliferative rate (as is indicated by the steepness of the growth curve in Figure 2A,B) compared to normal proliferative PAH ECFC and ECFC from control subjects.

#### 2.2.2. Relation to Markers of RV Function and Lung PVR

Proliferation was inversely related to RV end diastolic volume (RVEDV), indicating patients with highly proliferative ECFC were more likely to have preserved RVEDV (Figure 2C). No relation was found between proliferation and other parameters reflecting RV function (RVEF, CO) or between proliferation and PVR (Figure S4).

#### 2.2.3. Tube Formation

**Figure 3:**
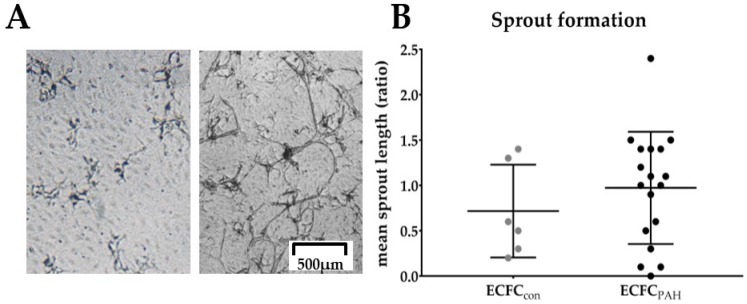
No difference in tube formation was observed among ECFC donors. (**A**) Representative phase contrast image of sprouts of PAH ECFC showing little or abundant tube formation. Scale bar indicates 500 μm. (**B**) Quantitative analysis of average tube length indicates no difference between control (grey, *n* = 6) or PAH ECFC (black, *n* = 18), normalized for mean tube length of control ECFC (*y*-axis, ratio). Mean with standard deviation (SD) is shown.

ECFC were seeded on fibrin matrices and stimulated with tumor necrosis factor (TNF-α), fibroblast growth factor (FGF) and vascular endothelial growth factor (VEGF) to induce tube formation (Figure 3A). Average length of tubes was quantified (Figure 3B). No difference was observed between ECFC derived from PAH patients and from control subjects, although notable inter-donor variation was present.

### 2.3. Transplantation of Highly Proliferative PAH ECFC with Tube Formation Ability in an Animal Model with Chronic RV-Failure

**Figure 4:**
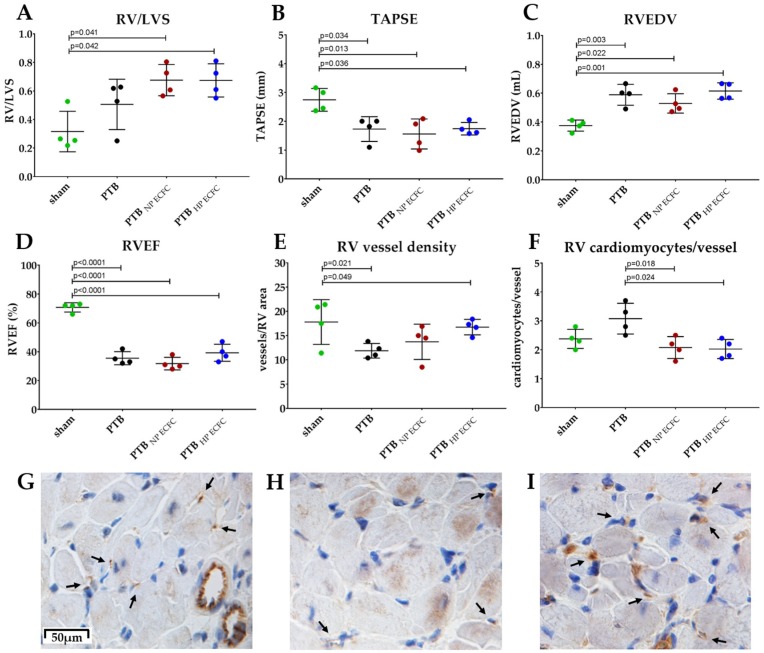
Transplantation of PAH ECFC did not induce reversal of the hemodynamic profile in PTB rats of chronic RV failure. (**A**) RV/LVS weight-ratio was comparable among sham rats (0.32 ± 0.14, green), PTB rats (0.51 ± 0.18, black), PTB rats transplanted with normal proliferative PAH ECFC (0.67 ± 0.11, red), or PTB rats transplanted with highly proliferative PAH ECFC (0.67 ± 0.11, blue). (**B**) TAPSE at evaluation shown of sham (2.7 ± 0.3 mm), PTB (1.7 ± 0.3 mm), PTB normal proliferative PAH ECFC (1.6 ± 0.5 mm), or PTB high proliferative PAH ECFC (1.7 ± 0.19 mm). (**C**) RVEDV of sham (0.38 ± 0.04 mL), PTB (0.59 ± 0.07 mL), PTB normal proliferative PAH ECFC (0.53 ± 0.07 mL) and PTB high proliferative PAH ECFC (0.62 ± 0.06 mL). (**D**) RVEF of sham (71 ± 3%), PTB (36 ± 5%), PTB normal proliferative PAH ECFC (32 ± 4%) and PTB high proliferative PAH ECFC (39 ± 6%). (**E**) Quantification of small vessel density/RV-area indicates significant reversal of the RV myocardial vessel density after transplantation with highly proliferative PAH ECFC (16.8 ± 1.6 vessels/RV-area), sham-animals (17.8 ± 4.6 vessels/RV-area), PTB (11.9 ± 1.5 vessels/RV-area), PTB normal proliferative PAH ECFC (13.7 ± 3.6 vessels/RV-area. (**F**) Cardiomyocytes per vessel ratio for sham (2.4 ± 0.4), PTB (3.1 ± 0.5), PTB normal proliferative PAH ECFC (2.0 ± 0.5) and high proliferative PAH ECFC (2.1 ± 0.6). Mean with SD is shown. Reported *p*-values are obtained from independent samples two way ANOVA with Bonferroni correction. (**G**–**I**) Representative phase contrast images of RV-muscle with brown CD31 staining for vessels (black arrow is pointed at a vessel) and nuclei (DAPI) of sham rats (**G**), PTB banded rats (**H**) and PTB banded rats transplanted with highly proliferative PAH ECFC (**I**). Scale bar indicates 50 μm.

Two PAH ECFC donors were selected for ECFC transplantation to rats: one donor with a highly proliferative and tube formation ability, and one donor with a normal proliferative and low sprouting ability. Highly and normally proliferative PAH ECFC were transplanted to rats with chronic RV failure induced by PTB. Two weeks after PTB, all animals showed signs of RV failure on echocardiography. Tricuspid regurgitation was present in all PTB animals, but not in sham animals. Tricuspid annular plane systolic excursion (TAPSE) was significantly lowered in PTB animals, as were stroke volume and cardiac output (Figure S5A–C). At evaluation, no difference was observed in RV/LVS ratio (Figure 4A). Echocardiography revealed a significantly lower TAPSE in PTB animals, but no difference in TAPSE between PTB ECFC transplanted groups (Figure 4B). Likewise, no differences in RVEDV or RVEF were observed on cardiac MRI other than between sham and PTB animals (Figure 4C,D). Catheterization showed no significant differences in right ventricular systolic pressure (RVSP), mean arterial pressure (MAP), end systolic elastance (Ees) or RV to arterial coupling (Ees/Ea) (Figure S6A–D). However, a significant increase in capillary density of the RV myocardium 3 weeks after transplantation was detected in PTB animals transplanted with highly proliferative ECFC (Figure 4E–I).Accordingly, cardiomyocytes/vessel ratio was lower in PTB rats after transplantation with highly proliferative ECFC (Figure 4F). Representative BF images of RV myocardium sections stained for CD31 are shown for sham and PTB animals (Figure 4G–I).

## 3. Discussion

To improve the understanding of the clinical significance of ECFC (dys-)function in PAH, we related ECFC function (i.e., blood outgrowth, tube formation, and proliferation) to markers of lung vascular remodeling and RV function. Our finding that blood-outgrowth of ECFC is more prominent in PAH patients with a severe clinical phenotype suggests that clinical severity is related to an increased recruitment of ECFC from the bone marrow. Accordingly, the presence of outgrowth was associated with lower RVEF, lower SvO_2_, and a shorter TTCW during 3 years of follow up. Somewhat paradoxically, in patients whose blood yielded ECFC, a high proliferative ability of these cells was related to preserved RV function, as indicated by a low RVEDV. Transplantation of highly proliferative ECFC to rats with severe RV failure induced reversal of vascular rarefaction but did not result in an improved RV function.

In our study, the blood of two thirds of PAH patients gave outgrowth to ECFC colonies. While selective outgrowth of ECFC in PAH was reported previously [12,14], our study is the first to relate presence of ECFC outgrowth to disease severity. ECFC are rare cells in the blood stream only consisting of 0.01–0.0001% of circulating mononuclear cells [25,26]. A percentage of 60–70% outgrowth of isolations is generally reported in health and disease [26,27,28]. Therefore, it was expected that not all PAH patients would yield ECFC. Interestingly, PAH patients with ECFC outgrowth were younger compared to PAH patients without ECFC outgrowth and were characterized by a significantly shorter TTCW and lower RV function. European registry studies (e.g., COMPERA and SPAHR) suggest that advanced age is an independent predictor for short transplantation-free survival, lower functional status and RV function, and diminished improvement on PAH targeted therapy [29,30]. These findings make it unlikely that age differences explain the differences in disease severity between PAH patients with and without ECFC outgrowth.

How disease severity influences the actual presence of ECFC in the bloodstream remains elusive. Recruitment of ECFC (and other EPC) from the bone marrow to the blood stream occurs upon release in tissues of bone-marrow targeting molecules, such as GM-CSF and SDF-1, in response to damage, inflammation or hypoxia [31,32]. It is thought that adherence and extravasation of ECFC play a role in the repair of injured tissue, as the transplantation of these cells leads to neovascularization of the infarcted area or site of endothelial damage [33,34]. The fact that we found outgrowth of ECFC in an exclusive subset of PAH patients may be due to differences in blood inflammatory and ECFC mobilizing factors [35,36,37] between PAH patients with mild or severe PAH disease [38]. These mediators may induce recruitment of ECFC from the bone marrow to the blood stream in order to induce tube formation and vascular repair [14].

We observed substantial inter-donor variation in proliferative rates of ECFC from patients with PAH. This finding confirms previous studies from Toshner et al. [13] and Asosingh et al. [12]. Likewise, we found heterogeneous sprout formation capacity of ECFC in our patient cohort. One previous study reported decreased sprout formation of ECFC from BMPR2 mutated PAH donors [12] and idiopathic PAH patients [18], but unaltered sprout formation of these cells in PAH was reported as well [15]. A cause of this heterogeneity may be that several signaling factors known to influence angio-proliferative function of ECFC, i.e., IL-6, IL1b, PDGF-b, and TNF-α [28], are highly upregulated in PAH. In vitro stimulation studies have shown that ECFC proliferation is strongly increased after stimulation with VEGF in concentrations of >50 ng/mL [39,40] and incubation with high concentrations of IL-1b (1–10 ng/mL) [41], while TNF-α [42] and low concentrated (0.25 ng/mL) IL-1b [41] are strong inhibitors of proliferation. Unfortunately, we did not measure levels of circulating cytokines in our patients.

While the proliferative rate of ECFC had no relationship with pulmonary vascular remodeling or PVR in our cohort of PAH patients, we found a moderate inverse relationship between proliferation rate of PAH ECFC and RV dilatation. One possible explanation for this finding is a direct hypertrophy and growth suppressing effect of inflammatory cytokines on both the RV and ECFC. For example, serum levels of TNF-α and IL-6, which are increased in PAH, have been attributed with cardio-depressive effects [43,44,45] as well as inhibitory effects on EC sprout formation and proliferation [46,47]. However, there is also an alternative explanation for the relationship between ECFC proliferative rate and RV function, namely that highly proliferative ECFC contribute to RV adaptation in PAH by stimulating myocardial revascularization.

Although we found no relationship between ECFC proliferative capacity and PVR, it remains possible that highly proliferative angio-proliferative ECFC contribute to pulmonary vascular remodeling and plexiform lesion formation. Accumulation of c-kit positive cells [48] and SDF-1 and its chemokine receptor CXCR4 [13] has been described in proximity of plexiform lesions while targeting of c-kit positive cells and CXCR-4 has been shown to ameliorate pulmonary hypertension in animal models [49,50]. Schiavon et al. [51] suggested that EPC accumulate in lung tissue of end stage PAH patients based on surface marker expression. The recent discovery of CD157 as a marker for tissue resident ECFC [52] may help expand the knowledge on this matter.

An imbalance in oxygen supply by cardiac vessels and oxygen demand by cardiomyocytes may lead to myocardial hypoxia, apoptosis, and contractile dysfunction in the development of hypertrophic cardiomyopathy [53]. Evidence of RV ischemia was provided by two independent nuclear imaging studies [54,55]. While possible explanations for RV ischemia include impaired coronary artery filling due to systemic hypotension and increased wall stress, a muscle-capillary mismatch has also been suggested.

Indeed, we observed that in a rat model of RV failure, transplantation of highly proliferative ECFC enhanced the capillary network in the RV. The fact that this increase in capillary density was not accompanied by an improvement in RV function could indicate that group sizes were too small, or that capillary rarefaction is not critical to the function of the pressure overloaded RV. Alternatively, the enhancement in capillarization could have been insufficient to result in functional improvement at the time of assessment. Whether repetitive transplantations and a longer follow-up would have resulted in more positive effects remains to be determined. In a follow-up study, stereology would be the best option to solidly prove increased myocardial revascularization.

In conclusion, we found donor dependent variation in outgrowth, proliferative, and tube formation ability of ECFC derived from patients with PAH. Outgrowth and proliferative capacity were related to markers of disease progression and RV-function. Transplantation of highly proliferative ECFC with tube formation ability reduced capillary rarefaction of the RV myocardium in rats after banding of the pulmonary trunk, although no improvement of RV function was observed. Restoring ECFC angio-proliferative ability could be a target for therapy.

## 4. Materials and Methods

### 4.1. Inclusion

WHO group 1.1, 1.2, and 1.4 PAH patients were recruited from the Pulmonary Hypertension clinic at the VU Medical Center, Amsterdam, The Netherlands. Control subjects were healthy volunteers as well as patients undergoing a right heart catheterization for diagnostic reasons but who were found to have normal hemodynamic values. The Institutional Ethical Review Board of the VU Medical Center (METC VU medical center) reviewed and approved the study (015.220-NL53211.029.15, approval-date 24th September 2015). Since the intervention consisted of a single blood draw, no signed informed consent was obligate before 7th of July 2017 by our Institutional Ethical Review Board. After this date, all patients gave informed consent. Comparison of baseline characteristics of PAH patients and control subjects are shown in Table S1.

### 4.2. Acquisition of Hemodynamic Parameters and RV Volume Measurements in PAH Patients

Right heart catheterization and pressure measurements were performed as described previously [56], and mean pulmonary arterial pressure (mPAP), pulmonary vascular resistance (PVR), cardiac output (CO), and central venous oxygen saturation (SvO_2_) were obtained from patient files. Magnetic resonance imaging was performed as described previously [56]. RV end diastolic volume (RVEDV) and RV ejection fraction (RVEF) were calculated from RV-cross-sectional images, excluding trabeculae. Only data within proximity of 1.5 year of ECFC isolation were included in this study.

### 4.3. Time to Clinical Worsening

Time to clinical worsening (TTCW) was obtained from patient records and defined by the time from ECFC isolation until death, emergency admittance to a hospital for re-evaluation or other cause attributable to PH, a decrease of ≥10% of 6 min walking distance (6MWT, or the start treatment with a prostaglandin-agonist (PGI2).

### 4.4. ECFC Isolation, Culture and Characterization

ECFC were isolated and cultured as described previously [28]. In short, the mononuclear cell fraction was isolated from whole blood using density centrifugation. Subsequently, cells were re-suspended in EGM-2 medium (EBM-2 with single quotes growth factor-kit, Lonza, Basel, Switzerland) supplemented with 10% human platelet lysate [28,57] and seeded on type I collagen- matrix (rattail collagen, BD, Franklin Lakes, NJ, USA). Culture plates were kept at 37 °C, 20% O_2_, 5% CO_2_. Culture medium was refreshed every other day. After 3 days, a washing step was performed to remove non-adherent cells. ECFC colonies appeared in culture after 7–28 days and were subsequently re-plated and expanded in a ratio of 1:5 until passage 4–7 was reached in order to perform experiments. Isolated cells were characterized as ECFC by presence of endothelial cell markers CD31 (555445, BD) VE-cadherin (561714, BD) VEGFR2 (5611714, BD) and lack of CD45 (557833, BD) using flow cytometry as previously described [58] (Figure S1).

### 4.5. Proliferation Assay

Proliferation was quantified over a period of 8 days. Primarily, ECFC were seeded in a density of 1000 cells/cm^2^ and cultured as described above. Donor specific proliferation curves were obtained by manual counting of cells/area from bright field microscopy pictures taken every day, with *t* = 1 being defined as 4 h after cell seeding. Proliferative speed (cells/day) was defined by the slope of the proliferation curve, obtained by linear regression of the linear part of the sigmoidal-shaped growth curve covering a minimum of 3 subsequent data points and *R*^2^ ≥ 0.97. PAH ECFC that exceeded the average proliferative speed of control ECFC by >2 SD were classified as being “high proliferative” PAH ECFC, while PAH ECFC donors that follow the growth curve of control ECFC were classified as “normal proliferative” PAH ECFC.

### 4.6. Tube Formation Assay

Sprouting ability was quantified from ECFC seeded in triplicate in a seeding density of 20,000 cells/cm^2^ on 3D human fibrin matrices and subsequent growth factor stimulation as described previously [28,58]. Following overnight incubation in M199 supplemented with 10% inactivated human serum and 10% newborn calf serum, tube formation was induced by stimulating the cells with combination of 10 ng/mL TNF-α, 10 ng/mL FGF-2, and 10 ng/mL VEGF (ReliaTech GmbH, Wolfenbuttel, Germany) in serum-supplemented medium every other day until sprouts of ECFC were visually detected. For quantification, ECFC were fixed with 2% paraformaldehyde/Hank’s Balanced Salt Solution (HBSS) and the length of formed sprout structures was quantified using semi-quantitative Optimas image analysis (Adapt Turkey, Perth, Australia).

### 4.7. Pulmonary Trunk Banding (PTB) in Rats

Male Wistar rats (Janiver Labs, Hannover, Germany) were treated according to the Danish National Guideline, and all experiments were approved by the Institutional Ethics Review Board and conducted in accordance with the Danish Law for animal research (authorization number 2016-15-0201-01040, Danish Ministry of Justice, Copenhagen, Denmark). Rats were housed two per cage with free access to water and standard rat chow (Altromin #1324; Altromin, Lage, Germany) in a room with a 12-h light-dark cycle and a temperature of 23 °C. At time of surgery, rats weighed 186 ± 28 g. PTB was performed as described previously by Andersen et al. [59]. Briefly, animals were randomized for PTB (*n* = 12) or sham-surgery (*n* = 2). All animals were anesthetized after which a lateral thoracotomy was performed and the pulmonary trunk carefully separated from the ascending aorta. In PTB animals, the banding was made with a horizon ligating clip applier modified to compress a titanium clip to a pre-set inner diameter of 0.5 mm around the pulmonary trunk.

### 4.8. Transplantation of PAH ECFC

Two weeks after PTB, baseline echocardiography was performed (Vevo 2100 echocardiographic system (Visual Sonics, Toronto, ON, Canada)) to assess tricuspid annular plane systolic excursion (TAPSE), and tricuspid regurgitation. After the echocardiography, PTB rats were randomized to transplantation with PAH ECFC of a highly proliferative donor with in vitro tube formation (*n* = 4), to transplantation with normal proliferative PAH ECFC with little tube formation (*n* = 4), or to saline injections (*n* = 4). Animals received transplantations via the tail vein at baseline and after 1.5 weeks after the first transplantation. Prior to transplantation, animals received an heparine bolus i.v. via a venflon in the tail vein, after which 1.5–2.0 × 10^6^ ECFC were administrated suspended in 0.4 mL warm 1% BSA/saline. Lines were flushed with heparinized saline. 2 sham and 2 PTB/saline animals were euthanized 1.5 weeks post sham/PTB procedures. All other animals were euthanized 3 weeks post-sham/PTB procedure. As no difference in hemodynamic, cardiac or anatomic data was observed, the 1.5 and 3 week time point post sham/PTB procedure were combined in order to increase power. All animals (sham and PTB) received daily subcutaneous injections with cyclosporine (15 mg/kg) to avoid ECFC rejection.

### 4.9. Acquisition of Hemodynamic Parameters and RV Volume Measurements in PTB-Animals

Prior to termination of the experiment, cardiac MRI was performed using a 9.4 Tesla Agilent MRI system. Imaging was synchronized with heart beat and respiration using a trigger system from SAII (New York, NY, USA). Measures of RV volumes were obtained from a series of cine short axis images, and cardiac output assessed using flow measurements from the pulmonary trunk obtained from a phase contrast sequence. Invasive pressure-volume measurement was performed using a small catheter (SPR-869, Millar Instruments, Houston, Texas, USA) installed in the left carotid artery and the RV. Slow occlusion of the inferior vena cava allowed simultaneous recordings of RV pressures and volumes with decreasing pre-load. Load independent measures of RV contractility, including end systolic elastance (Ees) and ventricular arterial coupling (Ees/Ea) were calculated from pressure volume-loops using LabChart Software (AD Instruments, Oxford, UK). Subsequently euthanasia was performed by extraction of the circulating blood volume, and organs were harvested.

### 4.10. RV-Small Vessel Density

RV tissue sections were deparaffinized and stained for CD31 (550300, BD, Franklin Lakes, NJ, USA) and subsequent DAB (BD, Franklin Lakes, NJ, USA) and H&E staining. Small, non-muscularized vessels were manually counted by two independent, blinded observers using BF microscopy-images from 6 randomly selected, transversal sections. RV tissue area was calculated with ImageJ software (National Institute of Health, Bethesda, MD, USA), and counted vessels were corrected for area.

### 4.11. Statistics

Continuous variables were visually inspected for normal distribution. If normally distributed mean with standard deviation (SD), and two-tailed independent student *t*-test or ANOVA was reported. Correlations were tested using univariate linear regression. For non-normally distributed data, median with interquartile range (IQR) and Mann-Whitney non-parametric test was reported. For dichotomous variables chi-quadrate was performed, or, in case of low numbers Fisher’s exact test. Cox-regression and −2log likelihood test were used for TTCW. A *p* value of ≤0.05 was considered significant. All tests were performed with SPSS^®^. IBM software, Armonk, NY, USA or GraphPad Prism 6^®^, GraphPad Software, La Jolla, CA, USA.

### 4.12. Handling of Missing Data

We choose a range of 1.5 year from time of ECFC isolation as a threshold for inclusion of hemodynamic and RV data. Because this is an observational study not all patients met this criterion. If so, the subjects were excluded from specific analysis.

## Figures and Tables

**Table 1:** Clinical characteristics of PAH patients with and without outgrowth of ECFC.

	ECFC Outgrowth	No ECFC Outgrowth	*p*
**Patient characteristics**			
*n*=	21	12	
Diagnosis (*n*)	iPAH(10), hPAH(5), PAH-CTD(3), PAH-CHD(2)	iPAH(7), hPAH(1), PAH-CTD(3), PAH-CHD(1)	
Age (yrs)	45 ± 12	56 ± 16	0.036 *
% female	90%	83%	
Disease duration (yrs)	4.1 (0.3–9.3)	2.7 (0.4–9.0)	0.687
Treatment PGI iv/sc (%)	52%	25%	0.122
**Laboratory tests**			
NT-proBNP	1296 (231–4441)	363 (154–888)	0.172
**Hemodynamics**			
mPAP (mmHg)	47 ± 18	47 ± 10	0.956
PVR (dynes.s.cm^−5^)	600 (383–928)	564 (214–882)	0.304
CO (l/min)	4.9 (4.1–6.1)	5.6 (3.8–9.0)	0.092
SvO_2_ (%)	63 (58–73)	72 (62–80)	0.037 *
**Cardiac function**			
RVEDV (mL)	171 (113–291)	145 (77–165)	0.238
RVEF (%)	29 (10–51)	46 (30-60)	0.037 *
**Performance**			
6MWT (m)	386 ± 156	381 ± 110	0.925
Heart failure acc. to NYHA	3.0 (2.0–3.5)	2.5 (2.0–3.9)	0.195
TTCW (mnths)	5.4 (0.6–29.2)	36.5 (7.4–63.4)	0.032 *
Transplantation list (%)	43%	33%	0.719
Death	40%	25%	0.443
FU (yrs)	3.0 (0.4–3.3)	3.6 (3.0–5.3)	0.687

iPAH, idiopathic PAH; hPAH, hereditary PAH; PAH-CTD, PAH associated with connective tissue disease; PAH-CHD, PAH associated with congenital heart disease; PGI, prostaglandin; NT-proBNP, *N*-terminal pro-brain natriuretic peptide; mPAP, mean pulmonary arterial pressure; PVR, pulmonary vascular resistance; CO, cardiac output; SvO_2_, mixed venous oxygen saturation; RVEDV, right ventricular end diastolic volume; RVEF, right ventricular ejection fraction; 6MWT, six minute walking test; TTCW, time to clinical worsening; FU, active follow up time from ECFC isolation until death or lung transplantation. Asterix denotes significance, defined by a *p*-value of 0.05.

## Supplementary Materials

Supplementary materials can be found at http://www.mdpi.com/1422-0067/19/12/3763/s1.

## References

[1] Voelkel N.F., Gomez-Arroyo J.G., Abbate A., Bogaard H.J., Nicolls M.R. (2012). Pathobiology of pulmonary arterial hypertension and right ventricular failure. Eur. Respir J..

[2] Guignabert C., Dorfmuller P. (2013). Pathology and pathobiology of pulmonary hypertension. Semin. Respir. Crit. Care Med..

[3] Vonk-Noordegraaf A., Haddad F.O., Chin K.M., Forfia P.R., Kawut S.M., Lumens J., Naeije R., Newman J., Oudiz R.J., Provencher S. (2013). Right Heart Adaptation to Pulmonary Arterial-Hypertension: Physiology and Pathobiology. J. Am. Coll. Cardiol..

[4] Gomez-Arroyo J., Sandoval J., Simon M.A., Dominguez-Cano E., Voelkel N.F., Bogaard H.J. (2014). Treatment for pulmonary arterial hypertension-associated right ventricular dysfunction. Ann. Am. Thorac. Soc..

[5] Rose J.A., Erzurum S., Asosingh K. (2015). Biology and flow cytometry of proangiogenic hematopoietic progenitors cells. Cytometry A.

[6] Ingram D.A., Mead L.E., Tanaka H., Meade V., Fenoglio A., Mortell K., Pollok K., Ferkowicz M.J., Gilley D., Yoder M.C. (2004). Identification of a novel hierarchy of endothelial progenitor cells using human peripheral and umbilical cord blood. Blood.

[7] Rehman J., Li J., Orschell C.M., March K.L. (2003). Peripheral blood “endothelial progenitor cells” are derived from monocyte/macrophages and secrete angiogenic growth factors. Circulation.

[8] Zhang H., Tao Y., Ren S., Liu H., Zhou H., Hu J., Tang Y., Zhang B., Chen H. (2017). Isolation and characterization of human umbilical cord-derived endothelial colony-forming cells. Exp. Ther. Med..

[9] Ikutomi M., Sahara M., Nakajima T., Minami Y., Morita T., Hirata Y., Komuro I., Nakamura F., Sata M. (2015). Diverse contribution of bone marrow-derived late-outgrowth endothelial progenitor cells to vascular repair under pulmonary arterial hypertension and arterial neointimal formation. J. Mol. Cell. Cardiol..

[10] Muller P., Beltrami A.P., Cesselli D., Pfeiffer P., Kazakov A., Bohm M. (2005). Myocardial regeneration by endogenous adult progenitor cells. J. Mol. Cell. Cardiol..

[11] Leone A.M., Valgimigli M., Giannico M.B., Zaccone V., Perfetti M., D’Amario D., Rebuzzi A.G., Crea F. (2009). From bone marrow to the arterial wall: The ongoing tale of endothelial progenitor cells. Eur. Heart J..

[12] Asosingh K., Aldred M.A., Vasanji A., Drazba J., Sharp J., Farver C., Comhair S.A., Xu W., Licina L., Huang L. (2008). Circulating angiogenic precursors in idiopathic pulmonary arterial hypertension. Am. J. Pathol..

[13] Toshner M., Voswinckel R., Southwood M., Al-Lamki R., Howard L.S., Marchesan D., Yang J., Suntharalingam J., Soon E., Exley A. (2009). Evidence of dysfunction of endothelial progenitors in pulmonary arterial hypertension. Am. J. Respir. Crit. Care Med..

[14] Zhu J.H., Wang X.X., Gu G.S., Shang Y.P., Zhang F.R., Chen J.Z. (2008). Reduced number and activity of circulating endothelial progenitor cells in patients with idiopathic pulmonary arterial hypertension. Respir. Med..

[15] Diller G.P., van Eijl S., Okonko D.O., Howard L.S., Ali O., Thum T., Wort S.J., Bedard E., Gibbs J.S., Bauersachs J. (2008). Circulating endothelial progenitor cells in patients with Eisenmenger syndrome and idiopathic pulmonary arterial hypertension. Circulation.

[16] Zhu J.H., Wang X.X., Zhang F.R., Shang Y.P., Tao Q.M., Zhu J.H., Chen J.Z. (2008). Safety and efficacy of autologous endothelial progenitor cells transplantation in children with idiopathic pulmonary arterial hypertension: Open-label pilot study. Pediatr. Transplant..

[17] Wang X.X., Zhang F.R., Shang Y.P., Zhu J.H., Xie X.D., Tao Q.M., Zhu J.H., Chen J.Z. (2007). Transplantation of Autologous Endothelial Progenitor Cells May Be Beneficial in Patients with Idiopathic Pulmonary Arterial Hypertension: A Pilot Randomized Controlled Trial. J. Am. Coll. Cardiol..

[18] Takahashi M., Nakamura T., Toba T., Kajiwara N., Kato H., Shimizu Y. (2004). Transplantation of endothelial progenitor cells into the lung to alleviate pulmonary hypertension in dogs. Tissue Eng..

[19] Yip H.K., Chang L.T., Sun C.K., Sheu J.J., Chiang C.H., Youssef A.A., Lee F.Y., Wu C.J., Fu M. (2008). Autologous transplantation of bone marrow-derived endothelial progenitor cells attenuates monocrotaline-induced pulmonary arterial hypertension in rats. Crit. Care Med..

[20] Xia L., Fu G.S., Yang J.X., Zhang F.R., Wang X.X. (2009). Endothelial progenitor cells may inhibit apoptosis of pulmonary microvascular endothelial cells: New insights into cell therapy for pulmonary arterial hypertension. Cytotherapy.

[21] Marsboom G., Pokreisz P., Gheysens O., Vermeersch P., Gillijns H., Pellens M., Liu X., Collen D., Janssens S. (2008). Sustained endothelial progenitor cell dysfunction after chronic hypoxia-induced pulmonary hypertension. Stem Cells.

[22] Aliotta J.M., Pereira M., Wen S., Dooner M.S., Del Tatto M., Papa E., Cheng Y., Goldberg L., Quesenberry P.J., Klinger J. (2017). Bone Marrow Endothelial Progenitor Cells Are the Cellular Mediators of Pulmonary Hypertension in the Murine Monocrotaline Injury Model. Am. J. Respir. Crit. Care.

[23] Zhao Y.D., Courtman D.W., Deng Y., Kugathasan L., Zhang Q., Stewart D.J. (2005). Rescue of monocrotaline-induced pulmonary arterial hypertension using bone marrow-derived endothelial-like progenitor cells: Efficacy of combined cell and eNOS gene therapy in established disease. Circ. Res..

[24] Wei L., Zhu W., Xia L.M., Yang Y., Liu H., Shen J.Q., Zhu J.S., Xu Y.W., Yang Z.H., Wang C.S. (2013). Therapeutic effect of eNOS-transfected endothelial progenitor cells on hemodynamic pulmonary arterial hypertension. Hypertens. Res..

[25] Siegel G., Fleck E., Elser S., Hermanutz-Klein U., Waidmann M., Northoff H., Seifried E., Schafer R. (2018). Manufacture of endothelial colony-forming progenitor cells from steady-state peripheral blood leukapheresis using pooled human platelet lysate. Transfusion.

[26] Khan S.S., Solomon M.A., McCoy J.P. (2005). Detection of circulating endothelial cells and endothelial progenitor cells by flow cytometry. Cytom. B Clin. Cytom..

[27] Kolbe M., Dohle E., Katerla D., Kirkpatrick C.J., Fuchs S. (2010). Enrichment of Outgrowth Endothelial Cells in High and Low Colony-Forming Cultures from Peripheral Blood Progenitors. Tissue Eng. Part C-Method.

[28] Tasev D., van Wijhe M.H., Weijers E.M., van Hinsbergh V.W., Koolwijk P. (2015). Long-Term Expansion in Platelet Lysate Increases Growth of Peripheral Blood-Derived Endothelial-Colony Forming Cells and Their Growth Factor-Induced Sprouting Capacity. PLoS ONE.

[29] Hjalmarsson C., Radegran G., Kylhammar D., Rundqvist B., Multing J., Nisell M.D., Kjellstrom B., SveFPH and SPAHR (2018). Impact of age and comorbidity on risk stratification in idiopathic pulmonary arterial hypertension. Eur. Respir. J..

[30] Hoeper M.M., Huscher D., Ghofrani H.A., Delcroix M., Distler O., Schweiger C., Grunig E., Staehler G., Rosenkranz S., Halank M. (2013). Elderly patients diagnosed with idiopathic pulmonary arterial hypertension: Results from the COMPERA registry. Int. J. Cardiol..

[31] Shahrivari M., Wise E., Resende M., Shuster J.J., Zhang J., Bolli R., Cooke J.P., Hare J.M., Henry T.D., Khan A. (2017). Peripheral Blood Cytokine Levels After Acute Myocardial Infarction IL-1 beta- and IL-6-Related Impairment of Bone Marrow Function. Circ. Res..

[32] Harbaum L., Renk E., Yousef S., Glatzel A., Luneburg N., Hennigs J.K., Oqueka T., Baumann H.J., Atanackovic D., Grunig E. (2016). Acute effects of exercise on the inflammatory state in patients with idiopathic pulmonary arterial hypertension. BMC Pulm. Med..

[33] Assmus B., Schachinger V., Teupe C., Britten M., Lehmann R., Dobert N., Grunwald F., Aicher A., Urbich C., Martin H. (2002). Transplantation of progenitor cells and regeneration enhancement in acute myocardial infarction-(TOPCARE-AMI). Circulation.

[34] Jiang S., Walker L., Afentoulis M., Anderson D.A., Jauron-Mills L., Corless C.L., Fleming W.H. (2004). Transplanted human bone marrow contributes to vascular endothelium. Blood.

[35] Li B., Sharpe E.E., Maupin A.B., Teleron A.A., Pyle A.L., Carmeliet P., Young P.P. (2006). VEGF and PlGF promote adult vasculogenesis by enhancing EPC recruitment and vessel formation at the site of tumor neovascularization. FASEB J..

[36] Shintani S., Murohara T., Ikeda H., Ueno T., Honma T., Katoh A., Sasaki K.I., Shimada T., Oike Y., Imaizumi T. (2001). Mobilization of endothelial progenitor cells in patients with acute myocardial infarction. Circulation.

[37] Kissel C.K., Lehmann R., Assmus B., Aicher A., Honold J., Fischer-Rasokat U., Heeschen C., Spyridopoulos I., Dimmeler S., Zeiher A.M. (2007). Selective functional exhaustion of hematopoietic progenitor cells in the bone marrow of patients with postinfarction heart failure. J. Am. Coll. Cardiol..

[38] Groth A., Vrugt B., Brock M., Speich R., Ulrich S., Huber L.C. (2014). Inflammatory cytokines in pulmonary hypertension. Respir. Res..

[39] Xie X.Y., Mo Z.H., Chen K., He H.H., Xie Y.H. (2011). Glucagon-like Peptide-1 improves proliferation and differentiation of endothelial progenitor cells via upregulating VEGF generation. Med. Sci. Monit..

[40] Yang L., Yang X.C., Yang J.K., Guo Y.H., Yi F.F., Fan Q., Liu X.L. (2008). Cyclosporin A suppresses proliferation of endothelial progenitor cells: Involvement of nitric oxide synthase inhibition. Intern. Med..

[41] Yang L., Guo X.G., Du C.Q., Yang J.X., Jiang D.M., Li B., Zhou W.J., Zhang F.R. (2012). Interleukin-1 Beta Increases Activity of Human Endothelial Progenitor Cells: Involvement of PI3K-Akt Signaling Pathway. Inflammation.

[42] Chen T.G., Zhong Z.Y., Sun G.F., Zhou Y.X., Zhao Y. (2011). Effects of tumour necrosis factor-alpha on activity and nitric oxide synthase of endothelial progenitor cells from peripheral blood. Cell Prolif..

[43] Lindner J., Maruna P., Kunstyr J., Jansa P., Gurlich R., Kubzova K., Zakharchenko M., Linhart A. (2009). Hemodynamic instability after pulmonary endarterectomy for chronic thromboembolic pulmonary hypertension correlates with cytokine network hyperstimulation. Eur. Surg. Res..

[44] Dolenc J., Sebestjen M., Vrtovec B., Kozelj M., Haddad F. (2014). Pulmonary hypertension in patients with advanced heart failure is associated with increased levels of interleukin-6. Biomarkers.

[45] Natanson C., Eichenholz P.W., Danner R.L., Eichacker P.Q., Hoffman W.D., Kuo G.C., Banks S.M., MacVittie T.J., Parrillo J.E. (1989). Endotoxin and tumor necrosis factor challenges in dogs simulate the cardiovascular profile of human septic shock. J. Exp. Med..

[46] Cuccuini W., Poitevin S., Poitevin G., Dignat-George F., Cornillet-Lefebvre P., Sabatier F., Nguyen P. (2010). Tissue factor up-regulation in proinflammatory conditions confers thrombin generation capacity to endothelial colony-forming cells without influencing non-coagulant properties in vitro. J. Thromb. Haemost..

[47] Huang P.H., Chen Y.H., Tsai H.Y., Chen J.S., Wu T.C., Lin F.Y., Sata M., Chen J.W., Lin S.J. (2010). Intake of Red Wine Increases the Number and Functional Capacity of Circulating Endothelial Progenitor Cells by Enhancing Nitric Oxide Bioavailability. Arterioscler. Thromb. Vasc. Biol..

[48] Montani D., Perros F., Gambaryan N., Girerd B., Dorfmuller P., Price L.C., Huertas A., Hammad H., Lambrecht B.N., Simonneau G. (2011). C-Kit-Positive Cells Accumulate in Remodeled Vessels of Idiopathic Pulmonary Arterial Hypertension. Am. J. Respir. Crit. Care.

[49] Gambaryan N., Perros F., Montani D., Cohen-Kaminsky S., Mazmanian M., Renaud J.F., Simonneau G., Lombet A., Humbert M. (2010). Targeting of c-kit+ hematopoietic progenitor cells prevents hypoxic pulmonary hypertension. Eur. Respir. J..

[50] Farkas D., Kraskauskas D., Drake J.I., Alhussaini A.A., Kraskauskiene V., Bogaard H.J., Cool C.D., Voelkel N.F., Farkas L. (2014). CXCR4 Inhibition Ameliorates Severe Obliterative Pulmonary Hypertension and Accumulation of C-Kit(+) Cells in Rats. PLoS ONE.

[51] Schiavon M., Fadini G.P., Lunardi F., Agostini C., Boscaro E., Calabrese F., Marulli G., Rea F. (2012). Increased tissue endothelial progenitor cells in end-stage lung diseases with pulmonary hypertension. J. Heart Lung Transplant..

[52] Wakabayashi T., Naito H., Suehiro J., Lin Y., Kawaji H., Iba T., Kouno T., Ishikawa-Kato S., Furuno M., Takara K. (2018). CD157 Marks Tissue-Resident Endothelial Stem Cells with Homeostatic and Regenerative Properties. Cell Stem Cell.

[53] Tomanek R.J. (1990). Response of the coronary vasculature to myocardial hypertrophy. J. Am. Coll. Cardiol..

[54] Vogel-Claussen J., Skrok J., Shehata M.L., Singh S., Sibley C.T., Boyce D.M., Lechtzin N., Girgis R.E., Mathai S.C., Goldstein T.A. (2011). Right and left ventricular myocardial perfusion reserves correlate with right ventricular function and pulmonary hemodynamics in patients with pulmonary arterial hypertension. Radiology.

[55] Van Wolferen S.A., Marcus J.T., Westerhof N., Spreeuwenberg M.D., Marques K.M., Bronzwaer J.G., Henkens I.R., Gan C.T., Boonstra A., Postmus P.E. (2008). Right coronary artery flow impairment in patients with pulmonary hypertension. Eur. Heart J..

[56] Spruijt O.A., Bogaard H.J., Vonk-Noordegraaf A. (2014). Assessment of right ventricular responses to therapy in pulmonary hypertension. Drug Discov. Today.

[57] Zeisberger S.M., Zoller S., Riegel M., Chen S., Krenning G., Harmsen M.C., Sachinidis A., Zisch A.H. (2010). Optimization of the culturing conditions of human umbilical cord blood-derived endothelial colony-forming cells under xeno-free conditions applying a transcriptomic approach. Genes Cells.

[58] Tasev D., Konijnenberg L.S., Amado-Azevedo J., van Wijhe M.H., Koolwijk P., van Hinsbergh V.W. (2016). CD34 expression modulates tube-forming capacity and barrier properties of peripheral blood-derived endothelial colony-forming cells (ECFCs). Angiogenesis.

[59] Andersen S., Schultz J.G., Andersen A., Ringgaard S., Nielsen J.M., Holmboe S., Vildbrad M.D., de Man F.S., Bogaard H.J., Vonk-Noordegraaf A. (2014). Effects of bisoprolol and losartan treatment in the hypertrophic and failing right heart. J. Card. Fail..

