# Supplementary material for: Blood Outgrowth and Proliferation of Endothelial Colony Forming Cells are Related to Markers of Disease Severity in Patients with Pulmonary Arterial Hypertension
Source: Int J Mol Sci. 2018 Nov 27;19(12):3763. doi: 10.3390/ijms19123763 (PMC6321271; doi:10.3390/ijms19123763)
Supplement: Supplementary file 1 [file ijms-19-03763-s001.pdf]

Supplementary materials

Supplementary table 1. Basic characteristics of control and PAH donors.

Table S1	PAH	Control
Number	21	20
Age	45±12	29±7
% female	91%	55%
Diagnosis (n)	HPAH (5);<br>IPAH (10);<br>PAH-CTD (3);<br>PAH-CHD (3);	n.a.
Disease duration (yrs)	7.7±4,9	n.a.
Heart failure acc. to NYHA	2.7±1,0	n.a.
PAH-specific treatment	95%	n.a.

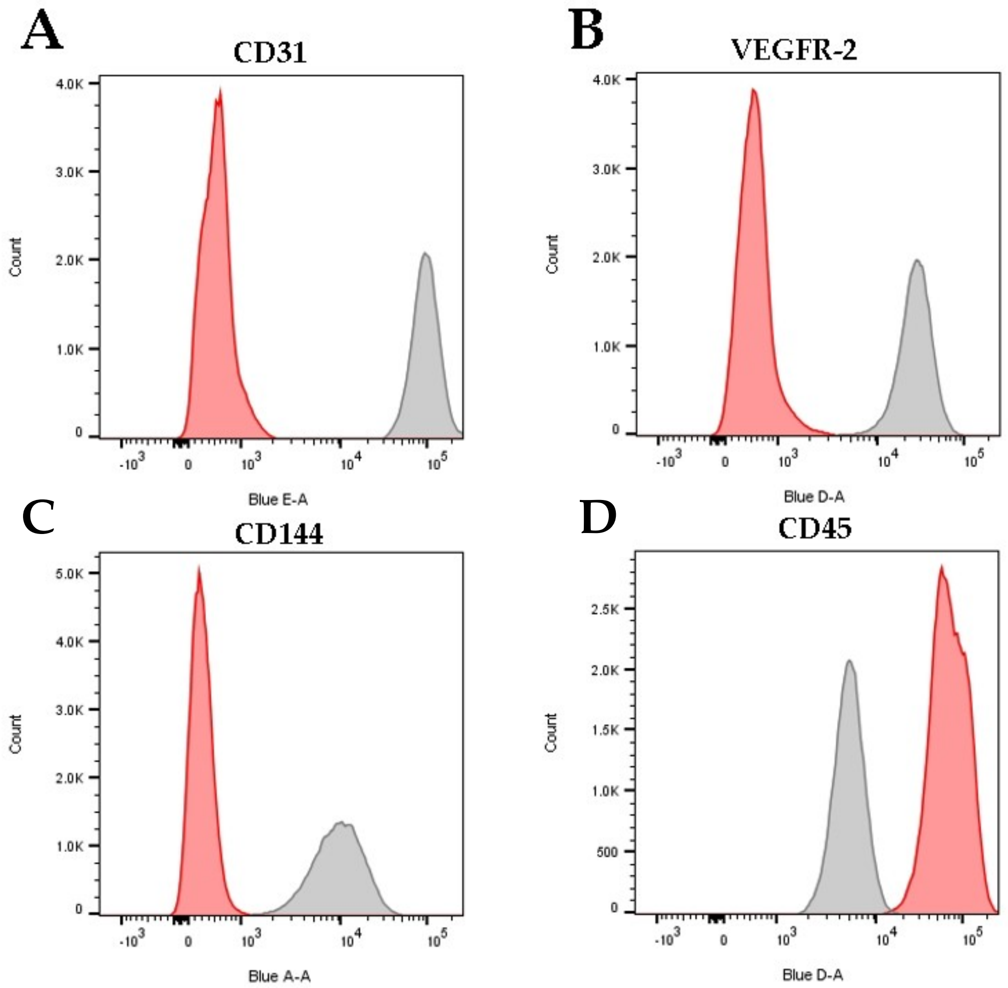

Supplementary figure S1 ECFC (grey) express mature EC markers CD31 **(A),** VEGFR-2 **(B)** and CD144 **(C),** but not CD45 **(D).** Macrophages were used as a negative control **(**red**, A-D)** and were CD45 positive **(D).**

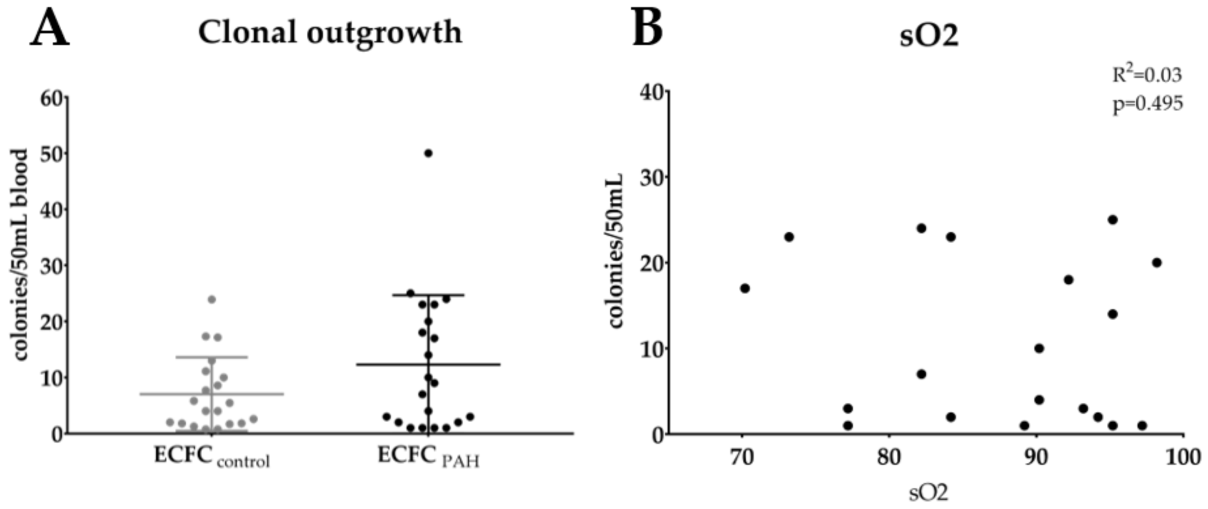

Supplementary figure S2 A) The quantity of single outgrown colonies of ECFC was equal among control and PAH donors. Control ECFC, n=20, 6.6±S1.5 colonies/50mL; PAH ECFC, n=21, 12,3±12,4 colonies/50mL blood; mean with SD are shown. **B)** proliferation of PAH ECFC (x-axis) versus peripheral oxygen concentration (sO2).

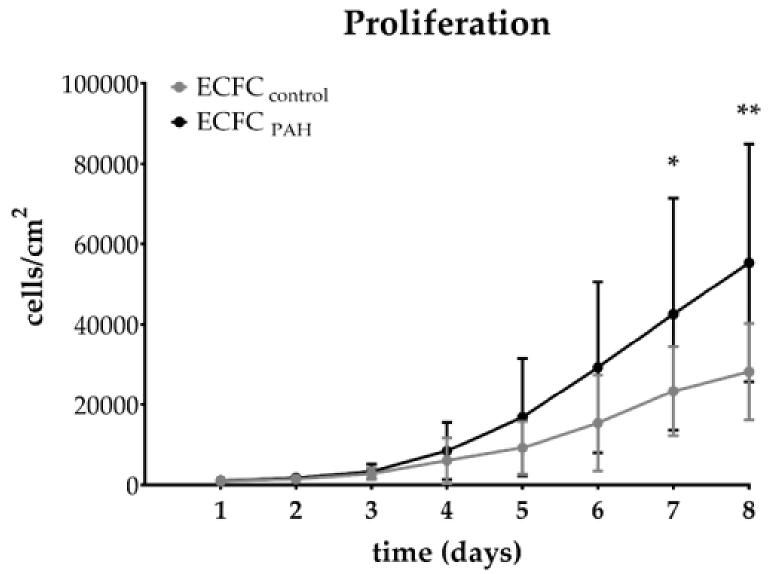

Supplementary figure S3 Merged proliferation curves of control ECFC (n=8) and PAH ECFC(n=18). Repeated measures ANOVA, p=0.018. Mean with SD is shown.

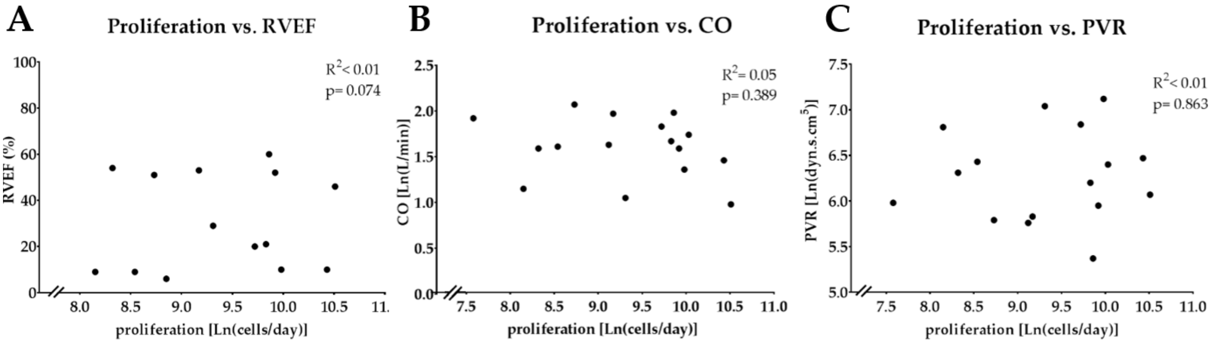

Supplementary figure S4 A) proliferation of PAH ECFC versus RVEF **(A),** CO; cardiac output **(B)** and PVR; pulmonary vascular resistance **(C).**

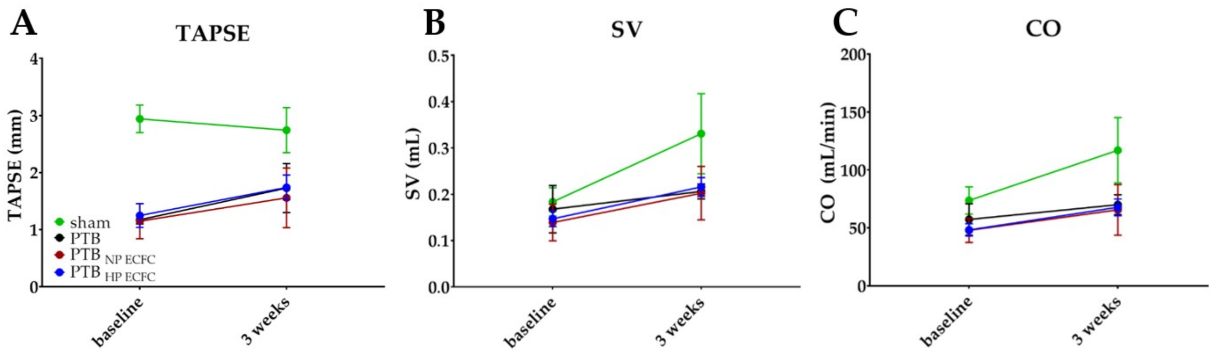

Supplementary figure S5 A) TAPSE of sham rats (green), PTB rats (blue) and PTB rats transplanted with normal proliferative (NP) PAH ECFC (red) or highly proliferative (HP) PAH ECFC (blue) at baseline and at the end of the experiment. **B)** SV at baseline and at the end of the experiment**. C)** CO, cardiac output at baseline and at the end of the experiment.

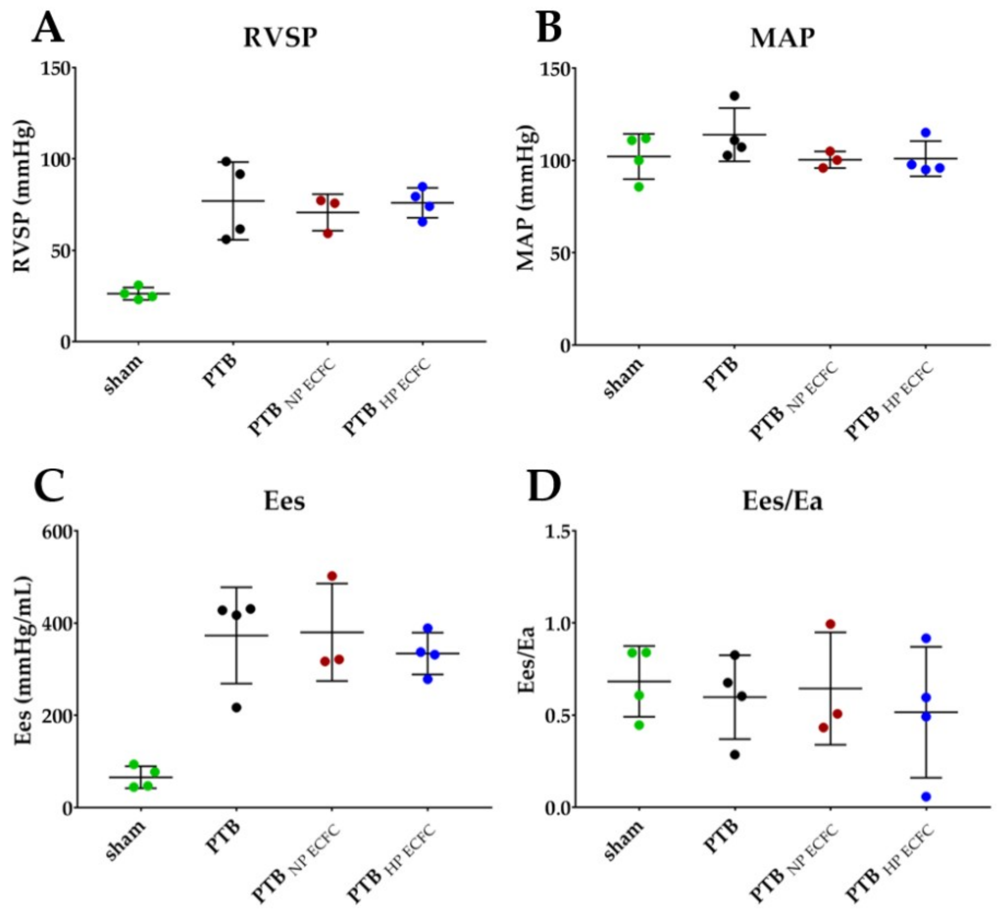

Supplementary figure S6 A) RVSP, RV end systolic pressure, for sham (green), PTB (blue) and PTB rats transplanted with normal proliferative (NP) PAH ECFC (red) or highly proliferative (HP) PAH ECFC (blue). **B)** MAP for all animals. **C)** Ees for all animals. **D)** Ees/Ea ratio of RV arterial coupling for all animals at evaluation.
